# Detection of the tuberculosis biomarker mannose-capped lipoarabinomannan in human serum: Impact of sample pretreatment with perchloric acid

**DOI:** 10.1016/j.aca.2018.09.037

**Published:** 2019-01-10

**Authors:** Nicholas A. Owens, Colin C. Young, Lars B. Laurentius, Prithwiraj De, Delphi Chatterjee, Marc D. Porter

**Affiliations:** a Department of Chemistry, University of Utah, Salt Lake City, UT, 84112, USA; b Department of Chemical Engineering, University of Utah, Salt Lake City, UT, 84112, USA; c Department of Bioengineering, University of Utah, Salt Lake City, UT, 84112, USA; d Department of Pathology, University of Utah, Salt Lake City, UT, 84112, USA; e Nano Institute of Utah, University of Utah, Salt Lake City, UT, 84112, USA; f Mycobacteria Research Laboratories, Department of Microbiology, Immunology and Pathology, Colorado State University, Fort Collins, CO, 80523, USA

**Keywords:** Tuberculosis, Lipoarabinomannan, Infectious disease diagnostics, Sample pretreatment, Point of need

## Abstract

The development of an accurate and rapid diagnostic test for tuberculosis (TB) to use at point of need is vital to efforts aimed at reducing the global burden from this disease. This paper builds on our previous studies of mannose-capped lipoarabinomannan (ManLAM) as a serum biomarker for active TB infection by means of a heterogeneous immunoassay. That work found that complexation with components in serum (*e.g.*, proteins) sterically hindered the capture and/or labeling of ManLAM in an immunoassay at levels <10 ng mL^−1^, compromising the clinical utility of this biomarker for detection of active TB infection. We also showed that the acidification of ManLAM-containing serum samples with perchloric acid improved the detectability of ManLAM by 250× by complex disruption when compared to measurements of untreated serum. The present study examined what effects the PCA treatment of serum samples may have on the recovery and structural integrity of ManLAM, owing to its potential susceptibility to acid hydrolysis. Recovery was assessed with an enzyme-linked immunosorbent assay (ELISA). The possible impact of acid hydrolysis on the ManLAM structure was investigated by gas chromatography-mass spectrometry and carbohydrate chemical degradation methods. The ELISA study indicated that while the signal strength for ManLAM in the serum spike-in experiments was significantly stronger after PCA pretreatment when compared to untreated human serum, it was only ∼20% of the ManLAM measured in physiological buffer. This loss in detectability was shown by structural analysis to arise mainly from the acid-induced degradation of the arabinan domains of ManLAM that are targeted by antibodies used for antigen capture and/or tagging. The implications of these findings in terms of the detection of this important biomarker for TB are also discussed.

## Highlights

• Tuberculosis (TB) is today’s deadliest infectious disease, largely due to lack of adequate diagnostic testing capabilities.
• Perchloric acid treatment decomplexes the TB marker mannose-capped lipoarabinomannan (ManLAM) from components in serum.
• Decomplexation improves ManLAM detectability in serum by 250×.
• The impact of acidification results in ManLAM degradation, lowering the efficiency of capture/labeling.
• Structural analysis data shows that this degradation is isolated to the arabinan domains of ManLAM.

## Graphical abstract

**Figure:**
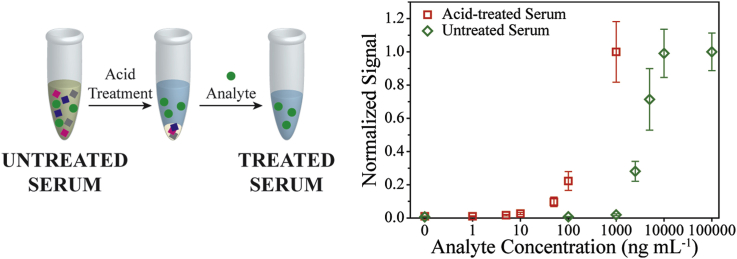

## 1 Introduction

The World Health Organization (WHO) estimates there were 1.3 million deaths associated with tuberculosis (TB) infections in 2016 [1]. While timely treatment often results in positive patient outcomes [1], diagnosing this disease early in its progression remains a technical barrier to reducing mortality. TB is especially burdensome in low and middle income countries (LMICs), where limited access to healthcare, economic barriers, and social stigma result in ∼40% of infected individuals remaining undiagnosed or unreported [[1], [2], [3], [4]]. These unfortunate circumstances place the development of a rapid, accurate, and low-cost test for TB that can be applied at the point of need (PON) as a vital step in reducing the burden associated with the disease [5,6].

The most used PON test for TB in LMICs is sputum smear microscopy, a reflection of its short turn-around time, low cost, and ease of use [7]. This test, however, requires an advanced stage of infection to be effective, which severely limits its utility for early stage diagnosis [7]. The gold standard for TB diagnosis is the bacterial culture of sputum samples [1,8]. While having a high clinical accuracy, this method has a turnaround time of a few weeks and requires an advanced laboratory infrastructure for implementation. Moreover, collecting sputum samples from children and some adults can be especially challenging, and sputum samples have questionable value in the diagnosis of extrapulmonary TB, which accounts for 10–15% of all TB cases [1,[9], [10], [11]]. Nucleic acid amplification tests (NAATs) also have a high level of clinical accuracy and have shown promise in detecting extrapulmonary TB, but high cost and procedural complexities continue to hinder PON applicability [[12], [13], [14], [15]].

In light of these challenges, a number of studies have focused on the development of tests using serum, urine, and other body fluids for the detection of ManLAM and other primary antigenic markers of *Mycobacterium tuberculosis* (Mtb), the causative agent of TB [[16], [17], [18], [19], [20], [21], [22], [23], [24], [25], [26]]. ManLAM is a lipoglycan (17.3 ± 5.0 kDa) that makes up ∼1.5% of the mycobacterium cell wall [[27], [28], [29], [30], [31]]. It has three main components: (1) a phospholipid anchor; (2) a mannan domain; and (3) an arabinan domain with varying degrees of branching and extent of capping motifs [[27], [28], [29],^32^]. The value of ManLAM as biomarker for TB arises from it being unique to mycobacteria as well as a major virulence factor in the infectious pathology of Mtb [33,34]. Moreover, ManLAM is readily shed from Mtb into the circulatory system, meaning the presence of ManLAM in serum should be directly linked to active infection [20,30,35,36].

**Fig. 1:**
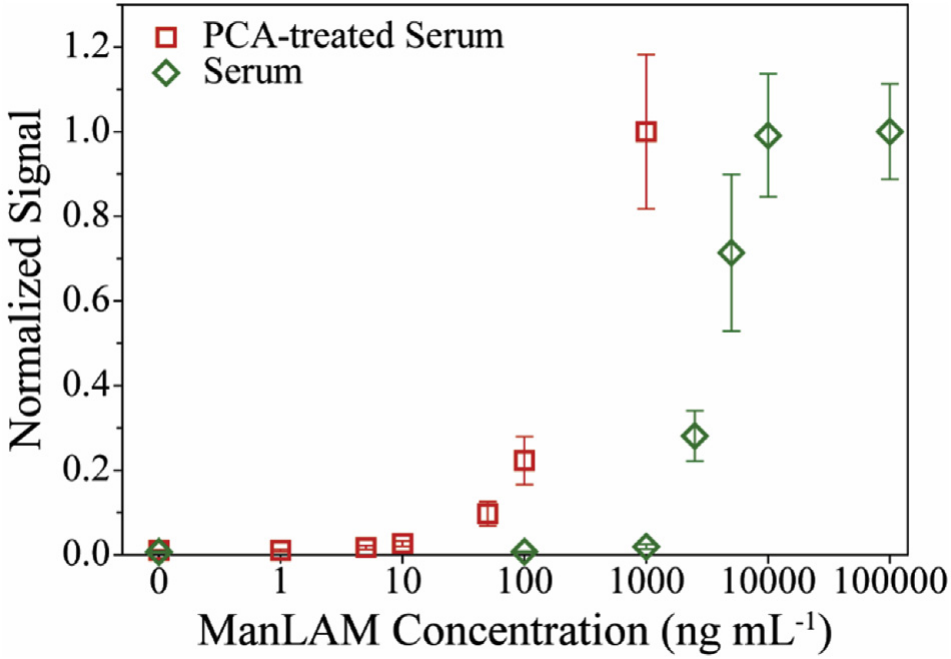
Dose-response plots (response normalized for each data set) for analysis of ManLAM directly from spiked human control serum (green) and for ManLAM spiked into human control serum and then PCA treated (red). These response-normalized plots were adapted using the data in Fig. 3, Fig. 4 in reference 42, which were obtained using a using an immunoassay read out with surface-enhanced Raman scattering (SERS). The LOD for the measurements on spiked serum without PCA treatment and with PCA treatment are 500 and 2 ng mL^−1^, respectively [42]. The average and standard deviation arise from triplicate assays, with each concentration run in triplicate for each assay. At lower concentrations, the error bars may be smaller than the graphical representation of the data points. (For interpretation of the references to colour in this figure legend, the reader is referred to the Web version of this article.)

Past studies on the clinical accuracy of ManLAM for TB diagnosis have reported high clinical specificities (*i.e.,* the reliability of a test to correctly identify a healthy patient as healthy), but widely varied and often low clinical sensitivities (*i.e.,* the reliability of a test to correctly identify a sick patient as sick) [37,38]. Along these lines, we recently found that the detectability of ManLAM in human serum significantly improved (∼250×) when analyzing the sample after acidification with perchloric acid (PCA) with an immunoassay as shown in Fig. 1 [[39], [40], [41], [42]]. We attributed the impact of acidification to arise, at least in part, from the tendency of ManLAM to complex with proteins and possibly other components in serum, and the resulting steric hindrance imposed by complexation on the antigen capture and/or tagging steps in the assay [42,43]. The addition of PCA lowers the pH of the sample, which induces protein denaturation and frees ManLAM for detection. These studies also showed that when applying PCA treatment to a set of TB patient specimens [24 TB-positive specimens (culture-confirmed), and 10 healthy controls], ManLAM was measurable in 21 of the 24 TB-positive specimens at levels ranging from 10 to 290 ng mL^−1^. ManLAM was not detectable [limit of detection (LOD) of 2 ng mL^−1^] in any of the healthy controls [42]. Taken together, these results begin to provide potential insights into the poor levels of clinical sensitivity reported in the earlier studies: the complexation of ManLAM sharply compromises its detection at low levels, which leads to a high false negative rate.

This work reports on ongoing efforts aimed at gaining insights into the possible pathways by which sample treatment improves ManLAM detection. The initial results from this study indicated that while PCA treatment improved ManLAM detection, the amount of ManLAM measured was actually ∼75% lower than the spiked-in levels. We therefore hypothesized that there were two possible origins for this low recovery: (1) capture surface passivation and/or the ineffective decomplexation of ManLAM from interferents in serum; (2) degradation of ManLAM due to its susceptibility to acid hydrolysis. The potential impact of each of these processes is investigated herein by using: (1) an in-house enzyme-linked immunosorbent assay (ELISA) to compare the detectability of ManLAM spiked into human serum, spiked human serum then PCA-treated, and spiked into physiological buffer, and (2) gas chromatography-mass spectrometry (GC-MS) and carbohydrate chemical degradation methods to assess the possible impact of acid hydrolysis on the structural integrity of the biomarker. The implications of these findings in terms of the reliable detection of this TB biomarker are discussed.

## 2 Material and methods

### 2.1 Materials and reagents

Dulbecco's phosphate buffered saline (PBS) buffer packs (pH 7.4 with 8 mM sodium phosphate, 2 mM potassium phosphate, 140 mM NaCl, and 2.7 mM KCl), StartingBlock (SB), and 1-Step 3,3ʹ,5,5ʹ-tetramethylbenzidine Ultra (TMB) were purchased from ThermoFisher Scientific. All buffers were prepared with 18.2 MΩ H_2_O, purified by a Barnstead ultrapure water system. Chloroform, Tween 20 (T20), 70% PCA, and potassium carbonate were acquired from Fisher Scientific. Trifluoroacetic acid (TFA), sodium borohydride, pyridine, acetic anhydride, 3-OMe glucose, bovine serum albumin (BSA), and concentrated sulfuric acid were obtained from Sigma Aldrich. Sulfuric acid (2N) was prepared by dilution with purified water. High affinity polystyrene 96-well microplates (Costar 3590) were purchased from Corning International. Pooled AB human male serum, hereafter referred to simply as human serum, was acquired from Innovative Research Inc. Streptavidin-modified horseradish peroxidase (HRP) and microplate sealers were from R&D Systems.

A VF-5ms (5% phenyl methylpolysiloxane) chromatography column was obtained from Agilent. ^13^C_5_-d-arabinose was purchased from Cambridge Isotope Laboratories. A polyclonal rabbit antibody (pAb) for Mtb, which served as the capture antibody, was used as received from Virostat (Cat. No. 4601). The human monoclonal antibody, mAb A194-01 for Mtb, was provided by Dr. Abraham Pinter at Rutgers University. ManLAM was isolated and purified by Dr. Delphi Chatterjee's laboratory at Colorado State University [29,44]. The use of these antibodies, which differ from those used in our earlier reports, reflects recent refinements in the assay soon to appear elsewhere [42,45]. Sulfo-NHS Biotin and Zeba Spin desalting columns, 0.5 mL sample volume with a molecular weight cutoff (MWCO) filter of 7 kDa, were obtained from Thermo Scientific. Additional Amicon 3 kDa MWCO filters were acquired from EMD Millipore. A biotin quantification kit was purchased from Pierce Biotechnology.

### 2.2 Enzyme-linked immunosorbent assay (ELISA)

The underlying principles and mechanisms of ELISAs have been reviewed in details elsewhere [[46], [47], [48], [49]]. The ELISA experiments were performed using high affinity 96-well microplates. To prepare a functionalized capture surface, pAb was diluted to 10 μg mL^−1^ in PBS, and 100 μL of the diluted solution was added to each well. The plates were sealed and incubated at 2–8 °C for ∼16 h to coat the wells with physisorbed antibody. The plate was then placed in a VorTemp™ incubator at a temperature of 30 °C and rotated at 250 RPM for 30 min. The capture antibody solution was removed by aspiration and the microwells rinsed. The aspiration and rinse steps were performed between each of the subsequent assay steps (blocking, antigen incubation, labeling, and HRP incubation) using a BioTek MultiFlo™ FX automated plate washer. Each rinse cycle was repeated three times using 300 μL of PBS with 0.05% Tween 20 (PBST) (v/v) at a flow rate of 422 μL/s (∼1.49 m/s). Next, 200 μL of SB was added to each well and incubated for 1 h in the VorTemp™ (30 °C and 250 RPM) in order to passivate uncoated portions of the polystyrene surface. All subsequent steps (antigen incubation, labeling, and HRP incubation) used the same rinsing procedure. ManLAM solutions for these experiments were prepared in PBS, PBS containing 1% BSA, or human serum with and without PCA. After preparation, 100 μL of the ManLAM-containing samples were added to the plate and incubated for 2 h.

Biotinylation of the A194-01 monoclonal antibodies followed the EZ-Link Sulfo-NHS-Biotin standard protocol provided by Thermo Scientific [50]. In brief, a 10 mM sulfo-NHS biotin solution was added at 50 times molar excess to a 1 mg mL^−1^ (6.7 × 10^−6^ M) solution of antibody and kept on ice for 3 h. Excess biotin was removed using a Zeba Spin desalting column by centrifuging at 1358 g for 1 min. The concentration of antibody and extent of biotinylation was determined to be 1.01 ± 0.01 mg mL^−1^ with a biotin-to-antibody ratio of 3.65:1, as measured using the Pierce biotin quantification kit. The modified A194-01 antibody was diluted to 200 ng mL^−1^ in PBS for the labeling step of the assay.

Captured ManLAM was labeled by adding 100 μL of the diluted tracer mAb to each well for 2 h. A 100 μL aliquot of streptavidin-HRP solution (diluted 1:200 in PBS) was then pipetted into each well and incubated for 25 min. Color development was performed by adding 100 μL of TMB to each well and incubating for an additional 25 min before the addition of 50 μL of a 2 N H_2_SO_4_ stop solution. The absorbance of the solution in the wells was immediately measured at 450 and 570 nm using a BioTek ELx800™ plate reader. The reported values are for the absorbance at 450 nm minus that at 570 nm. The limit of detection (LOD) for these assays is calculated as the average blank signal plus three times its standard deviation [39].

### 2.3 Acid treatment procedure

The PCA treatment of serum samples has been described in previous work [39,42]. In brief, the samples are acidified to pH ∼1 by adding 4 μL of PCA per 100 μL of sample in order to create denaturing conditions in the sample. The samples are then vortexed for 10 s and immediately centrifuged at ∼12,000 g for 5 min to pellet denatured proteins and other aggregated materials. A portion of the resulting clear supernatant containing the ManLAM is transferred to a clean microcentrifuge tube containing 9 μL of 2.0 M K_2_CO_3_ per 100 μL of starting sample volume to adjust the samples to pH ∼7.5. These samples are then stored at 2–8 °C for 60 min to accelerate precipitation of KClO_4_, after which the samples are centrifuged for 5 min at 270 g to ensure the precipitate has settled. The ManLAM-containing supernatant of each sample is transferred to a clean microcentrifuge tube and brought to ambient laboratory temperature prior to running the assay.

### 2.4 Carbohydrate analysis by gas chromatography-mass spectrometry (GC-MS)

To examine the potential degradation of ManLAM by PCA, an analysis was designed to test for the most likely ManLAM hydrolysis products (*i*.*e*., fragments from the arabinan side chain) [[26], [27], [28],[51], [52], [53], [54]]. For this, three ManLAM samples of 1.0 mL each, were prepared in PBS and divided into two separate 500 μL fractions. One fraction was then PCA treated. The other fraction was subjected to the same processing steps but with PBS replaced reagents specific to PCA treatment. Next, the samples were passed through a 3 kDa MWCO filter to separate intact ManLAM from any degradation products. This approach separates the degradation products below the 3 kDa cut off of the filter from those above the cut off, including intact ManLAM. In other words, the membrane retained intact ManLAM and the other fragments >3 kDa, while the smaller, more extensively degraded fractions passed through the membrane. The filtration step for each sample was repeated 4 times at 1398 g for 10 min. After the first filtration, the next three filtrations replenished the fluid phase by the addition of 200 μL of deionized water. The filtrate and the retentate after the final filtration were collected separately and lyophilized. ^13^C_5_-d-arabinose (1.0 μg), with 3-OMe glucose (1.0 μg) then added to each sample to serve as internal standards for arabinose and mannose, respectively.

The alditol acetate derivatization process used for the GC-MS analysis of arabinose and mannose and the method for using the internal standards have been detailed elsewhere [26,52]. Briefly, the samples undergo alditol acetate derivatization per the following steps: (1) trifluoroacetic acid [(TFA) 2 M] hydrolysis; (2) reduction with NaBH_4_; and (3) acetylation with pyridine and acetic anhydride. The samples are then partitioned between water and chloroform, and the organic layer, which contains the derivatized materials, is collected. Finally, the samples are dried under high purity N_2_(g) and reconstituted in 100 μL chloroform (HPLC grade) for analysis by GC-MS.

GC-MS analyses were carried out using a Varian CP 3800 gas chromatograph coupled to a MS320 mass spectrometer fitted with a J&W VF5ms (5% phenyl methylpolysiloxane) capillary column (30 m × 0.25 mm x 0.25 μm) column. The oven temperature upon injection was held at 100 °C for 1 min, and then ramped at 20 °C/min to 150 °C, 5 °C/min to 240 °C, and, finally, at 30 °C/min to 300 °C. The arabinose and the ^13^C_5_-arabinose internal standards have characteristic ions at *m/z* 217 and 220, respectively. The quantity of these two compounds was determined from the extracted ion chromatogram (EIC). The quantity of the mannose and 3-OMe glucose was determined using the total ion chromatogram (TIC). The amounts of arabinose and mannose, both of which are indicative of the amount of ManLAM in a sample, were determined from the intensity ratio of the *m/z* peaks for arabinose and mannose relative to those of ^13^C_5_-arabinose and 3-OMe glucose, respectively. Next, the peak ratios were multiplied by the mass of added ^13^C_5_-arabinose and 3-OMe glucose, which was 1000 ng for each. To account for the higher ion abundance of mannose relative to 3-OMe glucose, the resultant peak ratio was divided by a response factor of 1.44. Because ManLAM is composed of ∼60% arabinose and ∼40% mannose, the measured quantities of these two species can be used to calculate the original amount of ManLAM in the PCA-treated and untreated sample fractions [26,55,56].

## 3 Results and discussion

### 3.1 ManLAM recovery

As a starting point to characterizing the impact of PCA treatment on the detectability of ManLAM in human serum, the responses for three different sets of samples were measured: ManLAM-spiked untreated serum, ManLAM-spiked serum followed by PCA treatment, and, as a control, ManLAM-spiked PBS with 1% BSA (wt/v), which was used to mitigate nonspecific adsorption. Each sample type was prepared at 1000, 500, 100, and 50 pg mL^−1^ of ManLAM. Sample blanks consisted of stock human serum or PBS with 1% BSA devoid of ManLAM. The serum samples spiked with ManLAM were split into two equal volumes. One half of these samples was PCA treated; the other half of the samples added serum instead of the PCA reagents to reach the same dilution factor. The remaining steps were identical for both subsets. The two sets of samples prepared in PBS with 1% BSA were processed the same way, except PBS with 1% BSA was used in place of the PCA treatment reagents. Due to dilution from PCA treatment, the final ManLAM concentrations in the spiked samples after the treatment were 850, 425, 85.0, and 42.5 pg mL^−1^.

**Fig. 2:**
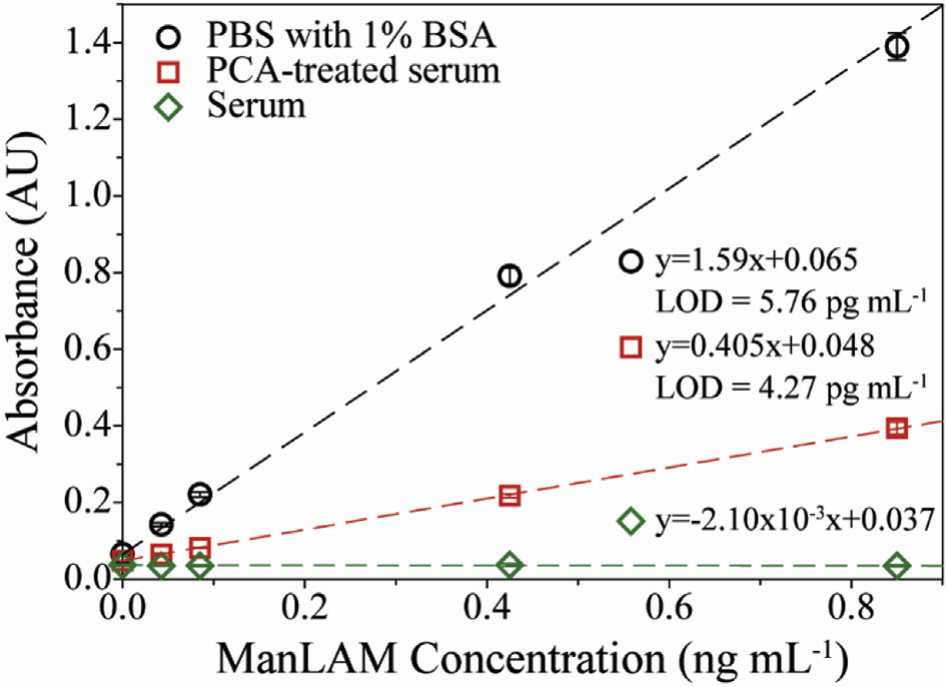
ELISA dose-response plots for ManLAM spiked into PBS containing 1% BSA (black), PCA-treated human serum (red), and serum only (green). The average and standard deviation arise from replicate assays (n = 5), with each concentration run in triplicate for each assay. At lower concentrations, the error bars are smaller than the graphical representation of the data points. (For interpretation of the references to colour in this figure legend, the reader is referred to the Web version of this article.)

Fig. 2 shows the ELISA response as a function of ManLAM spike-in concentration for the three different sample sets. There are two important points to draw from these plots. First, the difference in the responses for the PCA-treated serum samples and the untreated serum samples analyzed follow those presented in Fig. 1. Following the PCA treatment the measured signals are significantly increased reflecting the beneficial impact of acidification on the detectability of ManLAM.

Second, and of particular importance to the work reported on herein, the measurable responses for the PCA-treated serum samples are significantly lower than those for the ManLAM samples prepared in PBS with 1% BSA. In this experiment, after PCA treatment, the absorbance for ManLAM spiked into serum at 850 pg mL^−1^ is only 28% of that for the sample prepared in PBS with 1% BSA at the same spike-in level, indicating suboptimal ManLAM recovery in the PCA-treated samples. Five separate sets of these assays were run, with each run consisting of three tests at each sample concentration. These studies yielded an average recovery of 22 ± 11% for initial ManLAM concentrations between 85 pg mL^−1^ and 1.0 ng mL^−1^. The difference in assay performance is also evident from the fact that the analytical sensitivity (*i*.*e*., slope of the linear least squares calibration line) is a factor of ∼4 lower in the treated serum samples relative to the PBS with 1% BSA samples. Taken together, these results indicate that PCA treatment significantly improves ManLAM detection over that of untreated serum, but that the overall recovery is low and highly varied, leaving room for significant improvement.

As noted earlier, the lower response for the PCA-treated samples may be due to the passivation of the capture surface and/or the ineffective decomplexation of ManLAM. It is also possible that the susceptibility of furanosides to acid hydrolysis [[26], [27], [28],^51^] alters the structure of ManLAM in a way that degrades the epitopes targeted for antibody recognition [45]. The next sections investigate the possible contributions of each pathway.

### 3.2 Capture surface passivation

There are a massive number of components in human serum (*e.g*., proteins, carbohydrates, and electrolytes) that can potentially foul the capture surface and, therefore, interfere with ManLAM binding [[57], [58], [59]]. We previously showed that the total protein content remaining in control human serum after PCA treatment was 4% (2 mg mL^−1^) of that in as-received human serum (55 mg mL^−1^) [39]. In other words, there is still a significantly higher amount of protein present after acidification relative to the ManLAM spike-in levels. To determine if surface passivation plays a role in the measurements with PCA-treated serum, capture substrates were first blocked with PCA-treated serum or with whole serum. A set of substrates was also prepared by treatment with SB as a comparator. The three sets of wells were then exposed to ManLAM solutions prepared in PBS (1% BSA) at concentrations of 0, 500, and 1000 pg mL^−1^. The remaining steps of the assay were conducted as described previously.

**Fig. 3:**
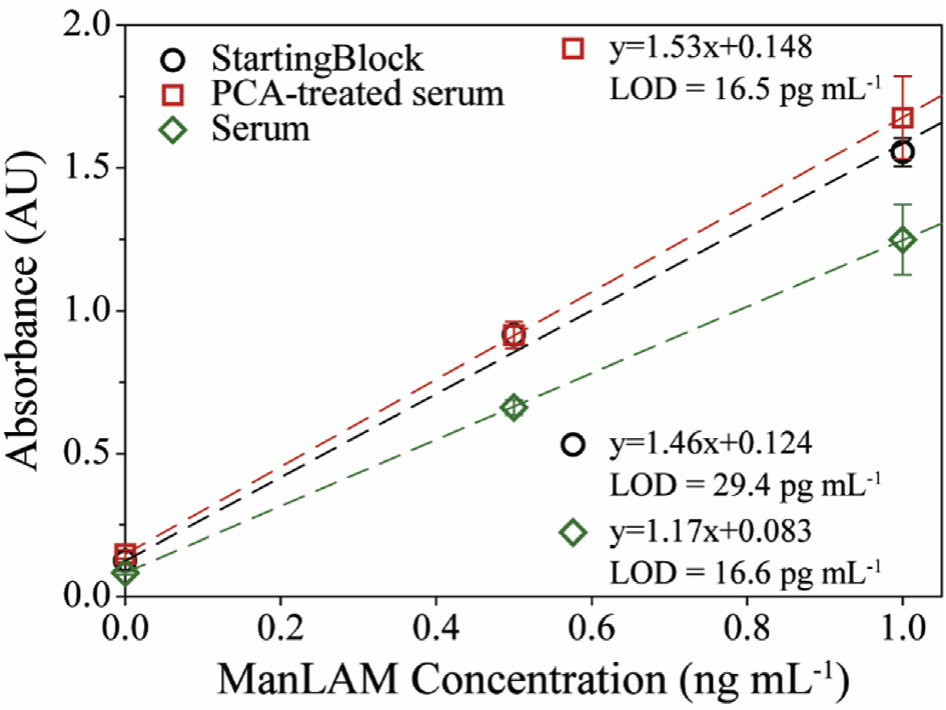
ELISA dose-response plots for capture substrates blocked with SB (black), PCA pretreated human serum (red), and untreated human serum (green). ManLAM solutions were prepared in PBS containing 1% BSA. The average and standard deviation arise from replicate assays (n = 3), with each concentration run in triplicate for each assay. For most samples, the error bars are smaller than the graphical representation of the data points. (For interpretation of the references to colour in this figure legend, the reader is referred to the Web version of this article.)

Fig. 3 presents the dose-response curves obtained for three sets of samples. The plot for the capture surface treated with SB is nearly indistinguishable from that in Fig. 2, which underscores the level of assay-to-assay reproducibility when using a comparatively innocuous sample matrix. In contrast, the responses for the assays when blocking with whole serum are lower than those with SB as a blocker. An average decrease of 24 ± 6% was observed for the 500 and 1000 pg mL^−1^ samples. Furthermore, the slope of the linear fit line when blocking with whole serum is 14% lower than that with SB blocking. This difference suggests that one or more of the components present in whole serum interfere with the ability of the capture antibody to effectively bind ManLAM. Note that this level of interference persists after completing all of the washing steps performed between the end of blocking and the step to capture ManLAM.

The PCA-treated serum samples, however, follow a response that is almost identical to that for ManLAM in PBS (1% BSA). The analytical sensitivities for the two dose-response plots are statistically indistinguishable using a Student's t-test at a 95% confidence interval. This result indicates that, unlike capture surfaces blocked with whole serum, the residual materials in the PCA-treated samples do not detectably passivate the capture surface or otherwise interfere with ManLAM capture. We can therefore conclude that surface passivation is not a measurable contributor to the diminished recovery of ManLAM from the PCA-treated serum samples, and that the reduction in signal reflects more important contributions from other factors.

### 3.3 Acid degradation of ManLAM

ManLAM has been found to be susceptible to acid hydrolysis, but the extent of the possible cleavage of its arabinan domain due to PCA treatment has not yet been examined in detail [28,60,61]. Epitope mapping studies indicate that the detection antibody is selective to the arabinan domain and mannose capping motifs of ManLAM [45,62]. As such, it is possible that any structural degradation of ManLAM could decrease or even eliminate the affinity interaction between ManLAM and the capture and/or labeling antibodies and therefore account for the decrease in the ELISA signal observed in Fig. 2 [63].

To determine if the PCA treatment process alters the structure of ManLAM, carbohydrate analysis by GC-MS was performed on as-prepared and PCA-treated samples of ManLAM spiked into PBS. We have recently used this methodology to measure the total ManLAM content in serum and urine samples in studies that have verified the utility of this marker for TB diagnostic testing [64]. These samples were prepared in PBS in order to avoid the endogenous arabinose found in human body fluids and to eliminate the tendency of the MWCO filter to clog upon passage of 1% BSA [26]. Our discussion of these results assumes that intact ManLAM remains in the retentate during the filtration step and that degradation products from acid hydrolysis pass through the 3 kDa MWCO filter (Section 2.4). By measuring the total arabinose and mannose content in all four sets of samples and by recognizing that ManLAM is composed of ∼60% arabinose and ∼40% mannose by weight, it is possible to determine the total amount of ManLAM originally present in the sample and to determine the extent, if any, in which PCA treatment degrades ManLAM [26]. The typical sample recovery factor and LOD (absolute mass) for this method for ManLAM is ∼95% and 500 pg, respectively [26].

Table 1 summarizes the results from the GC-MS analysis, which includes the measured amounts of arabinose and mannose in the filtrate and retentate for the PCA-treated and untreated samples. For the untreated samples, there was no detectable amount of arabinose or mannose in the filtrate. This indicates that, in the absence of PCA treatment, the structure of ManLAM is unaltered by the other solution processing steps. These results also serve as an internal check of the effectiveness of the overall measurement strategy in that the amount of ManLAM present in the retentate when comparing the measured levels of arabinose or of mannose are consistent across the two sample workups (see below).

**Table 1:** GC-MS results for three sets of treated and untreated ManLAM samples in PBS buffer.

	Untreated Samples		PCA-treated Samples	
	Retentate (ng)	Filtrate (ng)	Retentate (ng)	Filtrate (ng)
Arabinose	12.6 ± 3.9^b^	ND^a^	5.2 ± 0.2	8.6 ± 2.1
Calculated ManLAM from Arabinose	21.0 ± 6.5	ND	8.7 ± 0.3	14.1 ± 2.1
Mannose	9.2 ± 4.5	ND	9.0 ± 0.3	ND
Calculated ManLAM from Mannose	23.0 ± 11.2	ND	22.5 ± 0.8	ND

a ND: Not detectable (*i*.*e*., sample contains <500 pg).

b Errors represent the standard deviation of replicate samples (n = 3).

The results for the PCA-treated samples are much different. Arabinose is detectable in both the filtrate and retentate, but mannose is only measurable in the retentate. A little more than 60% (8.6 ± 2.1 ng) of the total measured arabinose in the PCA-treated samples is found in the filtrate; the remaining 40% (5.2 ± 0.2 ng) is in the retentate. This indicates that a significant portion of the arabinan domain is cleaved during the PCA treatment process. The cleaved arabinose components are most likely a mixture of monosaccharides and short (n = 2 or 3) oligosaccharides from the terminal ends of the side chains, which are further degraded to monosaccharides by TFA during the alditol acetate derivatization process [27,28,[52], [53], [54]]. Unlike arabinose, mannose is only detected in the retentate of both the PCA-treated and untreated samples, 9.0 ± 0.3 and 9.2 ± 4.5 ng, respectively. This indicates that (1) the core mannan structure remains intact during the PCA treatment process (*i.e.,* there is not a detectable level of mannose from either the core or capping motifs in the filtrate down to a detection level of ∼500 pg), and (2) the only detectable structural degradation occurs in the arabinan domain.

Based on the measured arabinose and mannose content, the calculated amounts of intact ManLAM in the retentate of the untreated samples were 21.0 ± 6.5 ng and 23.0 ± 11.2 ng, respectively. The calculated intact ManLAM content in the retentate and filtrate of the PCA samples was 8.7 ± 0.3 ng and 14.4 ± 2.1 ng, respectively, based on the measured arabinose content. The ManLAM content determined from the total arabinose detected in both the filtrate and retentate was 23.1 ± 1.9 ng. This is in good agreement with the calculated total ManLAM content, 22.5 ± 0.8 ng, based on the measured mannose found in the retentate. The agreement of the mass of mannose and arabinose in the treated and untreated samples is an indication of the reliability of the GC-MS analysis.

**Fig. 4:**
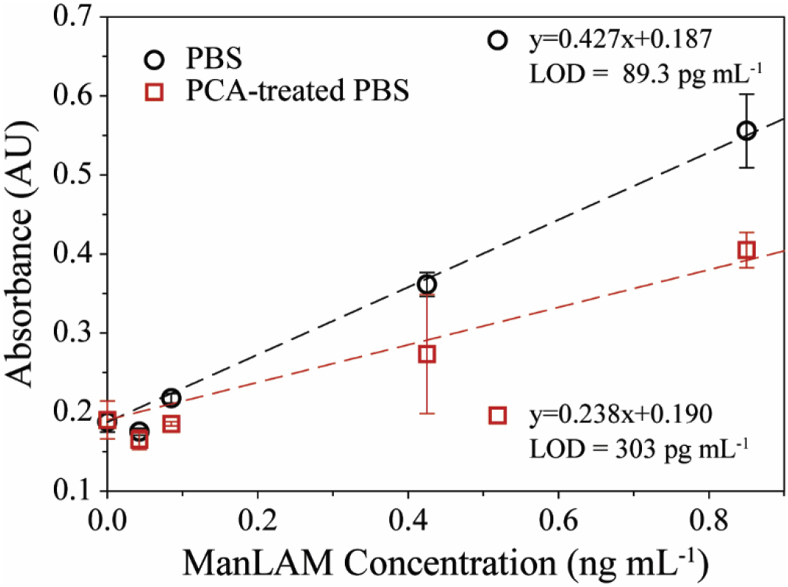
ELISA response for ManLAM spiked into PBS and for ManLAM spiked into PBS and then subjected to PCA treatment. The average and standard deviation arise from replicate assays (n = 3), with each concentration run in triplicate for each assay. For most samples, the error bars are smaller than the graphical representation of the data points.

To determine the effect of degradation on the ELISA measurements, a set of ManLAM dilutions were prepared in PBS and split into two equal volume fractions. Like the GC-MS experiments, one set of fractions underwent PCA treatment and the other had PBS added in place of the PCA treatment reagents in order to reach the same dilution factor. All remaining solution handling steps were the same for both fractions. These samples were prepared without BSA to remain consistent with the samples used for the GC-MS experiments. The ELISA dose-response plots from these measurements are presented in Fig. 4. The responses from the samples subjected to PCA treatment are much lower than those for the untreated samples. In this case, PCA-treated ManLAM in PBS samples at ManLAM concentrations ≥42.5 pg mL^−1^ have ∼23% reduction in signal sensitivity compared to the untreated samples. However, replicate assays (n = 5) have shown a loss of as much as 40% at ManLAM concentrations ≥ 42.5 pg mL^−1^ with an average of 26 ± 10%, highlighting how the extent of ManLAM degradation may impact immunorecognition with the antibodies on the underlying capture surface or during labeling [45].

Interestingly, the results in the ELISA sample sets differ from the analogous results for the GC-MS analysis by ∼10%. The GC-MS data, which represents a measure of the total ManLAM content in a sample, indicated that ∼62% of the ManLAM was degraded to some degree by PCA treatment. While the implications are not yet clear, these results suggest that the assay detects a portion of the partially degraded ManLAM. The ELISA results also support the conclusion that the degradation of ManLAM occurs primarily in the arabinan domain because the tracer antibody recognizes structural elements on the side chain, rather than at the mannan core [45]. Changes to the side chain structure of ManLAM can, therefore, result in a reduction of antibody recognition. This interpretation is consistent with a recent report that maps the antigenic heterogeneity of ManLAM with respect to the monoclonal antibody used in this work [45].

These results are notably different for those in Fig. 1, which shows a stronger reduction in signal when analyzing ManLAM-spiked serum samples after PCA pretreatment. We suspect this difference arises from the co-precipitation of ManLAM during the acid treatment process reducing its solution concentration. To date, however, our attempts to detect the presence of ManLAM by applying different extraction methods to the agglutinate have been unsuccessful. We are currently examining alternatives to this determination.

## 4 Conclusions

This work investigated the underpinnings of the use of a PCA treatment method as a means to liberate ManLAM from complexation in human serum that significantly improves its detection as a biomarker of active TB infection. Through ELISA studies, we showed that while PCA-treatment in human serum improved detectability, the amount of ManLAM recovered was only ∼20% of the spike-in levels. Moreover, structural analysis investigations with GC-MS indicated that the susceptibility of ManLAM to acid hydrolysis resulted in the degradation of the arabinan domains of the biomarker, which had a negative impact on antigen recognition in our assay. This work highlights the importance of designing comprehensive sample treatment strategies that can counter the impact of the sample matrix on detection while also taking into account the potential impact of treatment on the analyte. Ongoing work is focused on fully assessing how to improve both the amount and reproducibility of ManLAM recovery by acidification, the use of other chaotropic agents, and the merits of enzymatic treatment methods using proteinase K and other proteases [45].

## Declarations of interest

None.

## Funding

This work was supported by the Critical Path Initiative of the U.S. Food and Drug Administration (contract U18 FD004034) and the Bill and Melinda Gates Foundation (OOP10396210). CCY acknowledges support from the National Science Foundation Graduate Research Fellowship Program (Grant Number 1256065).
